# Are Antioxidants a Potential Therapy for FSHD? A Review of the Literature

**DOI:** 10.1155/2017/7020295

**Published:** 2017-06-12

**Authors:** Adam Philip Denny, Alison Kay Heather

**Affiliations:** Department of Physiology, School of Biomedical Sciences, University of Otago, Dunedin, New Zealand

## Abstract

Facioscapulohumeral muscular dystrophy (FSHD) is an inherited myopathy affecting approximately 1 in 7500 individuals worldwide. It is a progressive disease characterised by skeletal muscle weakness and wasting. A genetic mutation on the 4q35 chromosome results in the expression of the double homeobox 4 gene (DUX4) which drives oxidative stress, inflammation, toxicity, and atrophy within the skeletal muscle. FSHD is characterised by oxidative stress, and there is currently no cure and a lack of therapies for the disease. Antioxidants have been researched for many years, with investigators aiming to use antioxidants therapeutically for oxidative stress-associated diseases. This has included both natural and synthetic antioxidants. The use of antioxidants in preclinical or clinical models has been largely successful with a plethora of research reporting positive results. However, when translated to clinical trials, the use of antioxidants as a therapeutic intervention for a variety of disease has been largely unsuccessful. Moreover, specifically focusing on FSHD, limited research has been conducted on the use of antioxidants as a therapy in either preclinical or clinical models. This review summarises the current state of antioxidant use in the treatment of FSHD and discusses their potential avenue for therapeutic use for FSHD patients.

## 1. Introduction

Facioscapulohumeral muscular dystrophy (FSHD) is an inherited myopathy characterised by progressive muscle weakness and wasting. FSHD is the third most common muscular dystrophy [1–3], estimated to affect 1 in 7500 individuals worldwide. As indicated by the name of the dystrophy, the facial, humeral, and shoulder girdle muscles are most significantly affected; however, the foot extensors, rectus abdominis muscles, and pelvic-girdle muscles can also become affected towards the latter stage of the disease progression [4]. Individuals with FSHD experience muscle weakness and atrophy, which is often asymmetric [5]. Unlike other muscular dystrophies, the pharyngeal muscles and cardiac muscles are rarely affected in FSHD [4]. Cardiac involvement is found in approximately only 5% of individuals with FSHD and when present manifests as a predisposition to atrial arrhythmias [6]. Around 1% of individuals with FSHD experience restrictive respiratory disease which requires an intervention; however, this is typically seen in those FSHD patients with severe muscle weakness [7]. Patients can also present with additional symptoms such as severe muscular inflammation, subclinical hearing loss, and abnormalities of the peripheral retinal capillary [8–12]. Research has also suggested that FSHD can result in changes to the central nervous system. For instance, Quarantelli et al. [13] reported that patients with FSHD demonstrated a lower volume of grey matter, which was slightly correlated to clinical severity (*P* < 0.05); however, no correlation was found between brain tissue volume and D4Z4 repeat size. Research has also shown links between FSHD, mental retardation, and epilepsy, with patients presenting with a larger deletion in the FSHD region having the greatest possibility of displaying severe clinical phenotypes with central nervous system abnormalities [14, 15].

## 2. Genetics of FSHD

FSHD is the result of an epigenetic mechanism which results in disturbances to the transcriptional control of multiple genes [16]. There are two different forms of FSHD, FSHD1 and FSHD2. Of those who are diagnosed with FSHD, 95% of the individuals are diagnosed with FSHD1 with the remainder being FSHD2. FSHD1 is due to deletions in the subtelomeric region chromosome 4q35 within an array of D4Z4 repeat elements. FSHD2, which presents with the same symptoms as FSHD1, is due to a mutation on chromosome 18 resulting in deficiency of the protein SMCHD1 which normally interacts with the D4Z4 repeat on chromosome 4 [17]. As FSHD1 is the most common form of FSHD, the scope of this review is limited to the FSHD1 condition only. FSHD1, which hereafter will be referred to as FSHD, is characterised by a shortened D4Z4 repeat array which consists of 1–10 repeats, whereas in healthy individuals the repeat units range from 11 to 150 [18]. Individuals can present with a similar D4Z4 shortening which is located on chromosome 10q; however, no contractions of the 10qter have been reported to result in FSHD [19, 20].

**Figure 1:**
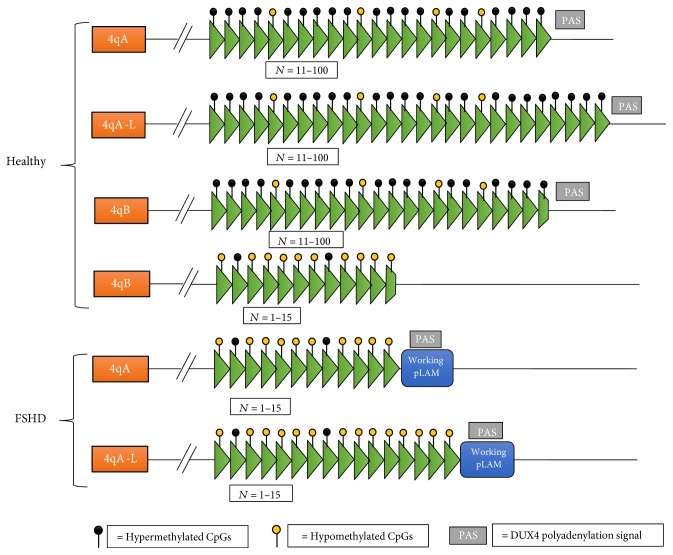
The molecular signatures of chromosome 4 in both FSHD and healthy individuals. Healthy unaffected individuals carry 11–100 repeat units, whereas individuals with FSHD carry less than 15 repeat units (green triangles represent repeat units). Contraction of the D4Z4 repeat in FSHD results in the protein DUX4 being expressed but only in conjunction with a 4qA allele and working pLAM. Individuals with FSHD have an increased amount of hypomethylated CpGs which also contributes to the transcription of DUX4.

The D4Z4 repeat array within FSHD individuals contains the double homeobox 4 gene (DUX4), and contractions of the D4Z4 repeat array results in epigenetic changes leading to the expression of DUX4 [21, 22]. DUX4 is active during early development but is silenced by hypermethylation of the D4Z4 region, such that in healthy adult cells, DUX4 is not produced. Within FSHD, the shortened region leads to hypomethylation which allows the expression of DUX4. However, hypomethylation of the D4Z4 region only results in FSHD when it occurs with a permissive chromosome 4. There are two allelic variants of the 4q subtelomere which are both just as common as each other in a healthy population; these are named 4qA and 4qB [23, 24]. FSHD is only associated with the 4qA allele which contains a shortened D4Z4 repeat and working pLAM sequence which allows the abnormally shortened DUX4 gene to produce protein [23]. A similar repeat on the 4qB-type allele will not cause FSHD [25]. The main difference between the 4qA- and 4qB-type alleles is the interstitial (TTAGGG)*_n_* array adjacent to the 68 bp satellite DNA within 4qA [24]; (Figure 1). Furthermore, in some instances, the 4q D4Z4 repeat array has been shown to contain an extra 2 kb of D4Z4 sequence, which results in a longer repeat unit in *cis* with a 4qA allele; this is commonly referred to as the 4qA-L allele [26]; ( Figure 1).

## 3. Clinical and Molecular Pathophysiology

**Figure 2:**
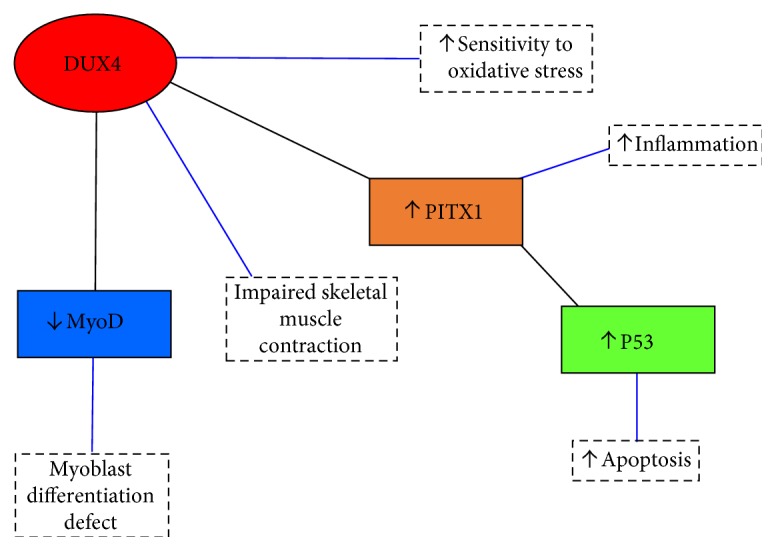
Schematic of the transcriptional cascade effect of DUX4 in which it increases sensitivity to oxidative stress, inflammation, and apoptosis; impairs skeletal muscle contraction; and results in a myoblast differentiation defect. PITX1 = pituitary homeobox 1; P53 = tumour protein 53; MyoD = myogenic differentiation.

As previously mentioned, contractions of the D4Z4 repeat array results in DUX4 expression. The DUX4 protein is a transcription factor which drives expression of gene products that mediate the cell inflammatory response ([10]; Figure 2). It is also responsible for a large gene deregulation cascade and inducing genes involved in oxidative stress, inflammation, toxicity, and skeletal muscle atrophy [27–29]. Beyond its direct role as a transcription factor, DUX4 has an indirect role on gene expression because it regulates pituitary homeobox 1 (PITX1; [22, 30–32]). Importantly, PITX1 induces expression of the tumour protein 53 gene (P53; [29, 32]). Overexpression of P53 is orchestral in FSHD as it promotes skeletal muscle atrophy by increasing inflammatory cytokine levels, which subsequently exacerbate further oxidative stress [28, 29, 32]. In addition to the increase in inflammation and oxidative stress, DUX4 expression results in a decrease in MyoD expression (myogenic differentiation), which plays a major role in regulating skeletal muscle differentiation [33].

## 4. Oxidative Stress and FSHD

**Figure 3:**
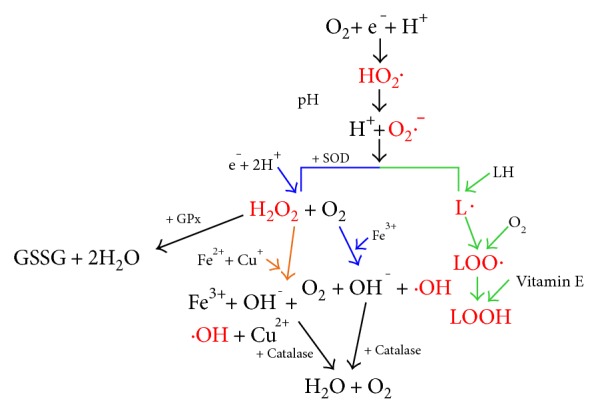
A schematic of the reactions which lead to the formation of free radicals. Red text indicates free radicals. Green arrows represent lipid peroxidation, orange arrows represent the Fenton reactions, and the blue arrows represent the Haber-Weiss reactions. SOD is the enzyme superoxide dismutase, and GSSG refers to glutathione disulphide. HO_2_· = hydroperoxyl radical; O_2_·^−^ = superoxide radical; H_2_O_2_ = hydrogen peroxide; ·OH = hydroxyl radical; L· = lipid radical; LOO· = fatty acid peroxyl radical; LOOH = lipid hydroperoxide.

Oxidative stress is often referred to as a condition where the increased production of free radicals or reactive oxygen species (ROS) and oxidative-related reactions results in cellular damage [34]. Examples of free radicals include superoxide (O_2_·^−^), hydroxyl (·OH), nitric oxide (NO·), and peroxynitrite (ONOO^−^). O_2_·^−^ is the most common free radical in the body, and it occurs as a result of an oxygen molecule obtaining an unpaired electron, thus becoming negatively charged and unstable (Figure 3). Multiple processes can result in an oxygen molecule becoming charged and unstable, with examples being the oxidation of the catecholamines and the activation of arachidonic acid cascade [35]. The cellular enzymes NADPH oxidase and xanthine oxidase (XO) are known to be major contributors in the production of O_2_·^−^. Although O_2_·^−^ is a radical, it is not very reactive [34] and the antioxidant enzyme, superoxide dismutase (SOD), converts O_2_·^−^ to the weaker oxidant, hydrogen peroxide (H_2_O_2_). H_2_O_2_ is relatively stable in its natural state; however, in the presence of transition ions such as iron and copper, it has the ability to rapidly diffuse across cell membranes and convert into ·OH radicals [35]. ·OH radicals have an extremely short half-life and are one of the most potent oxidants in the body. Notably, they react at their site of formation and attack most biological molecules, which in turn propagates free radical chain reactions [34, 35].

The production of O_2_·^−^ is not just limited to enzymatic reactions. Under certain conditions, mitochondria and the electron-transport chain will produce ROS [36]. When the concentration of oxygen within the mitochondria decrease, O_2_·^−^ and ·OH radicals are produced [36]. Moreover, the production of O_2_·^−^ is not just limited to oxygen concentration; it is also reliant on the concentration of potential electron donors [37]. Complex I is known to drive significant O_2_·^−^ production within the mitochondria, particularly in two conditions: firstly, when there is a high proton motive force due to insufficient ATP production and a reduced coenzyme Q (CoQ) pool [38, 39] and secondly, when the mitochondrial matrix has a high NADH/NAD^+^ ratio [40, 41]. The production of mitochondrial ROS can disturb cellular homeostasis through driving redox signalling, mitochondrial dysfunction, and apoptosis/necrosis.

Antioxidant enzymes such as SOD, glutathione peroxidase (GPx), and catalase are involved in protecting cells from being damaged by ROS [42]. As previously stated, SOD converts O_2_·^−^ to the weaker radical H_2_O_2_. H_2_O_2_ can then be broken down by GPx in the mitochondria and cytosol in a reaction where glutathione (GSH) is oxidised producing glutathione disulphide and 2H_2_O [43]. H_2_O_2_ can also be broken down into H_2_O and O_2_ by the enzyme, catalase (Figure 3). Overproduction of ROS can pose a serious problem to bodily homeostasis because of oxidative damage to tissues [44]. These adverse effects of ROS can be limited by the natural antioxidant pathways represented by SOD, GPx, and catalase. Moreover, as will be discussed in more detail later, under certain circumstances, if ROS becomes too high, natural antioxidant pathways can become overwhelmed, allowing excess ROS to cause cellular damage [44].

Individuals with FSHD have been reported to have an altered oxidative stress response, in part due to differential expression of proteins [33, 45–49]. FSHD myoblasts were shown to have increased levels of the protein p21, when compared to healthy myoblasts under normal growth conditions [49]. Increased levels of p21 have previously been shown to be upregulated in human and murine fibroblasts in response to oxidative stress [50]. p21 is known to induce cell cycle arrest [51], and it has been reported that p21 expression correlates with the onset of muscle differentiation in myoblasts growing under normal conditions [52]. Winokur et al. [49] concluded that due to the increased p21, FSHD myoblasts wrongly signal a state of oxidative stress to their nucleus, resulting in cell cycle arrest, hindering the formation of new myotubes. Furthermore, Tassin et al. [53] reported that SOD levels were increased in FSHD-affected muscle, along with two other proteins involved in the oxidative stress response, glutathione *S*-transferase and thioredoxin peroxidase. These three proteins act to protect against oxidative stress, and it is hypothesised that within FSHD, the antioxidant defence system is increased to try and alleviate the increased oxidative stress. In addition to changes in the antioxidant defence system and oxidative stress signalling, the oxygen storage in FSHD muscle is also affected [47]. It was reported that levels of myoglobin (a protein found in the muscle which binds to iron and oxygen, thus storing oxygen in the muscles) in FSHD muscle were 25% lower than those in healthy muscle. The increases reported in oxidative stress response proteins are likely a compensatory mechanism for the increased ROS levels and decreased oxygen storage capacity in FSHD muscle.

FSHD muscle shows marked changes in mitochondrial metabolism through a decrease in cytochrome *c* oxidase and increases in NADH-dehydrogenase flavoprotein and NADH-ubiquinone oxidoreductase [47]. This is expected to be the results of the decreased oxygen storage capacity of the muscle; thus, mitochondrial dysfunction occurs resulting in an increase in oxidative stress. Turki et al. [54] reported that mitochondria organisation in FSHD human skeletal muscle biopsies was at times markedly altered when compared to healthy controls, with large mitochondria pools in the intermyofibrillar and subsarcolemmal compartments. Furthermore, Turki et al. [54] reported that FSHD patients have increased oxidative stress, increased inflammatory response, and abnormal mitochondrial function when compared to healthy controls. Both mitochondrial dysfunction and oxidative stress correlated with function muscle impairment [54].

Excessive oxidative stress and inflammation of the skeletal muscle can interfere with muscular contractions. In mammalian skeletal muscle, excitation contraction coupling depends on motor neuron-induced cell depolarization and the subsequent interaction between dihydropyridine receptor (DHPR) and the ryanodine receptor (RyR1), which results in Ca^2+^ being released from the terminal cisternae of the sarcoplasmic reticulum [55]. RyR1s are redox-sensitive channels; therefore, alterations in redox state can result in their activation [56, 57] or inactivation [58]. Furthermore, Stoyanovsky et al. [56] reported that in under oxidative stress, RyR1s can become unstable resulting in an increased open probability that subsequently causes Ca^2+^ leak. Along with the oxidative modification of RyR1, pro-oxidant states lead to the activation of CaMKII [59]. The activation of CaMKII has previously been shown by Rodney et al. [60] to promote the phosphorylation of RyR1, which consequently results in the leaky release of Ca^2+^ from the sarcoplasmic reticulum [55].

More specifically, in regard to muscular dystrophy, Bellinger et al. [61] reported that mdx mouse muscle (a mouse model of Duchenne muscular dystrophy (DMD)) experiences Ca^2+^ leak as a result of cysteine-nitrosylation, which is likely to contribute to muscle weakness within the disease. DMD is the result of a deficiency in dystrophin which severely disrupts the dystrophin glycoprotein complex (DPG), leading to muscle damage as the result of pathological Ca^2+^ signalling [62–64]. The mdx mouse model has a nonsense point mutation on the dystrophin gene, from glutamine to threonine, resulting in the production of a nonfunctioning dystrophin protein [65]. Although the mdx mouse is the most commonly used model of DMD, it is often seen as a “milder” form of DMD as they present minimal clinical symptoms and a lifespan reduced by 25% [66] in comparison to a reduction of 75% in humans. Moreover, Andersson et al. [67] reported that RyR1 channels in a murine model of limb-girdle muscular dystrophy (LGMD; a deficiency in *β*-sarcoglycan—a protein involved in the DPG) muscle are oxidised, cysteine nitrosylated, phosphorylated, and depleted of calastabin1 which results in leaky channels along with a decreased fast twitch muscle force and impaired capacity for exercise. The same authors [67] went on to suggest that leaky RyR1 channels may underline multiple forms of muscle dystrophy, particularly those which have a disrupted DPG and suggested that pharmaceutical targeting of RyR1 channels may be a potential therapeutic treatment in DMD and LGMD. To date there is no known link between Ca^2+^ leak and FSHD; however, based on the data presented in DMD and LGMD, it is hypothesised that Ca^2+^ leak may be a pathological mechanism in FSHD.

## 5. Synergy between Oxidative Stress and Inflammation

In addition to oxidative stress, FSHD individuals experience increased skeletal muscle inflammation which can also interfere with muscular contraction. Muscle atrophy as a result of chronic inflammation is associated with increased proinflammatory cytokine production, such as tumour necrosis factor alpha (TNF-*α*), interleukin 1 (IL-1), interleukin 6 (IL-6), and interferon gamma (IFN-*γ*; 59). The overexpression of TNF-*α* has been shown to result in significant body and muscle mass loss in mice [68, 69]. Moreover, Li et al. [70] reported that cultured C2C12 skeletal muscle myotubes show protein degradation following prolonged exposure to TNF-*α* (1–6 ng · ml^−1^). It was reported that TNF-*α* stimulates protein degradation through binding to surface receptor (TNFR1), which induced mitochondrial production of ROS resulting in a rapid activation of the ubiquitin-proteasome pathway and the degradation of I-*κ*B*α*, the protein responsible for inhibiting the key inflammatory response mediator NF-*κ*B [70]. As a result, NF-*κ*B is activated, translocates to the nucleus to switch on expression of its target genes, including a subset that controls muscle proteolysis. Linking together oxidative stress and inflammation, Kosmidou et al. [71] reported that C2C12 myotubes exposed to either XO or H_2_O_2_ for 24 hours showed a concentration-dependent increase in IL-6 production. In regard to FSHD specifically, Turki et al. [54] reported that patients with FSHD presented with significantly increased levels of the proinflammatory cytokines TNF-*α* (*P* < 0.001), IFN-*α*2 (interferon alpha-2; *P* < 0.001), RANTES (regulated on activation, normal T-cell expressed and secreted; *P* < 0.001), MCP1 (monocyte chemotactic protein-1; *P* < 0.01), and IL-6 (*P* < 0.01), along with a significant positive correlation between TNF-*α* and glutathione disulphide (GSSG) levels (*P* = 0.02). To date, this is the only known data which shows a synergistic relationship between oxidative stress and inflammation in FSHD; thus, it remains to be defined in any real detail.

In vivo, similar mechanisms have been shown to operate with proinflammatory cytokines inducing muscle wasting via the activation of the ubiquitin-proteasome pathway and apoptosis [72–74]. Baracos et al. [72] reported that the soleus and extensor digitorum of young male CD-strain mice incubated in IL-1 for two hours experienced a significant increase in protein degradation. The authors reported that proteolysis increased; however, the rate of protein synthesis remained the same. Furthermore, the incubation of muscles in IL-1 also resulted in the synthesis of prostaglandin E_2_, which is known to augment muscle protein breakdown.

In addition to these biochemical changes to protein, oxidative stress can lead to impaired muscle contractions because of effects on muscle force. Callahan et al. [75] reported that O_2_·^−^ and ·OH significantly decreased maximum calcium-activated force by 14.5% and 43.9%, respectively, in rat diaphragm fibres. Furthermore, Edwards et al. [76] reported that mouse extensor digitorum longus (EDL) untreated muscle exposed to O_2_·^−^ significantly decreased maximum Ca^2+^-activated specific force (kN/m^2^; *P* < 0.001); however, this was attenuated when the muscle was pretreated with the potent synthetic antioxidant, Tempol (4-hydroxy-2,2,2,2-tetramethylpiperidine-1-oxyl). When the muscles were incubated at 37 °C with 1 mM Tempol, they showed no significant reduction in contractile capacity, suggesting that antioxidants such as Tempol can reduce the levels of oxidative stress. These results explain that the contractile capacity of mouse muscle is impaired by the superoxide anion radical, likely due to oxidation of various contractile chemical groups, particularly those located on the myosin heads. Together, these findings show the vulnerability of FSHD muscle to oxidative stress, leading to a reduction in contractile capacity and ultimately muscle weakness in addition to showing the potential of antioxidants to help alleviate the problems caused in FSHD.

## 6. Antioxidants and FSHD

As oxidative stress underlies FSHD, antioxidant treatment is a potential therapeutic option. El Haddad et al. [77] reported that healthy and FSHD muscles pretreated with retinoic acid (RA; a metabolite of vitamin A) presented lower ROS levels and apoptosis than when exposed to H_2_O_2_ overnight (both *P* < 0.05). Additionally, myoblast adhesion was restored by pretreatment with RA and associated with a higher IC50 (the H_2_O_2_ concentration at which half of the cells adhere to the culture dish). The underlying protective mechanism involved glutathione peroxidase 3 (GPx3) showed significantly higher GPx3 mRNA levels (*P* < 0.01) in healthy human myoblasts pretreated with RA, when compared to untreated human myoblasts. In addition, GPx3 activity was significantly increased following exposure to RA (*P* < 0.05). Limited clinical trials have been performed studying the effects of vitamin A supplementation; thus, it is unclear if it would be useful clinically, especially for FSHD patients.

Synthetic antioxidants have been shown to reduce oxidative stress in FSHD muscle, along with slowing disease processes. Bosnakovski et al. [78] identified 52 compounds which can inhibit DUX4-induced toxicity in myoblasts. Several of these compounds were known to be antioxidants, with the authors reporting that a large amount of the other compounds lacked any obvious reducing activity; therefore, it was hypothesised that these antioxidants protect cells from oxidative stress in an indirect manner. Furthermore, Dmitriev et al. [79] reported that FSHD myoblasts present increased DNA damage. This is evident by high levels of the DNA damage marker protein *γ*H2AX (*P* < 0.05; the phosphorylation of histone H2A variant H2AX at Ser139) and increased apurinic/apyrimidinic sites (abasic site; a location of the DNA which has neither a purine or pyrimidine base, this may occur spontaneously or are a result of DNA damage). If DUX4 levels are knocked down via siRNA in FSHD primary myoblasts, the DNA damage was significantly reduced (*P* < 0.05). Together, these findings provide clear evidence that DUX4 induces DNA damage. If the FSHD myoblasts were treated with Tempol, there was reduced DNA damage (*P* < 0.05). However, DNA damage was not reduced completely. Tempol was also shown to improve myotube formation of FSHD myoblasts. Similar findings were obtained with the synthetic antioxidant NAC (N-acetylcysteine). Therefore, antioxidant treatment may reduce DNA damage in FSHD myotubes, thus improving muscle health.

To date, a plethora of research has shown that exercise can induce antioxidant effects and alleviate oxidative stress. It is known that exercise results in an acute increase in ROS; however, this is shortly followed by a compensatory increase in antioxidant defences [80]. Gomez-Cabrera et al. [81] reported that exhaustive exercise conducted in rats results in the activation of mitogen-activated protein (MAP) kinases and the NF-*κ*B pathway which in turn stimulates the production of antioxidant defences such as SOD. When rats were administered with allopurinol (an inhibitor of XO which is involved in O_2_·^−^ production following exhaustive exercise), the adaptive changes in antioxidant defences and muscle adaptations were abolished, thus confirming that exercise can be considered as a potent antioxidant. Furthermore, Karabulut et al. [82] reported that following a 12-week aerobic exercise program, the lipid peroxidation marker malondiadehyde (MDA) significantly decreased whereas the antioxidant parameters SOD and GSH significantly increased. More recently, a systematic review and meta-analysis conducted by de Sousa et al. [83] reported that physical activity induced antioxidant effects and decreased pro-oxidant effects regardless of the type of exercise, volume, intensity, and population studied. Consequently, it can be concluded that exercise is antioxidant in nature and can balance the redox state to elicit positive health outcomes.

Antioxidants act to decrease oxidative stress and reduce the threat free radicals pose to bodily homeostasis. However, in certain situations, antioxidants can become pro-oxidants and will elicit the opposite effect to what is desired. Pro-oxidants can induce oxidative stress via two pathways, firstly through the production of free radicals and secondly, through inhibiting the antioxidant defence systems. For example, vitamin C is primarily considered a potent antioxidant, but when it is combined with iron and copper, it promotes a reaction which results in iron or copper being reduced from Fe^3+^ to Fe^2+^ or Cu^3+^ to Cu^2+^, respectively. These augment the reduction of H_2_O_2_ to ·OH thereby increasing ROS. Similarly, *α*-tocopherol (vitamin E), another well-known antioxidant, can become pro-oxidant when used at high concentrations. This occurs because upon reacting with a free radical, *α*-tocopherol becomes the tocopheroxyl radical, and without the presence of ascorbic acid, it will remain in this reactive state. It has been shown that the tocopheroxyl radical promotes the autoxidation of linoleic acid [84].

The well described antioxidants, carotenoids, can also become pro-oxidants in certain situations. At high concentrations, carotenoids exposed to oxidative stress undergo oxidative breakdown which drives pro-oxidative effects through the formation of ·OH radicals [85, 86]. Similarly, flavonoids can act as pro-oxidants and when they are in the presence of both transition ions such as copper, iron or zinc, and oxygen. In such an environment, flavonoids can be catalysed resulting in the formation of ROS and free radicals such as the phenoxyl radical [87]. Phenoxyl radicals primarily result in DNA and lipid damage.

Some synthetic antioxidants have also been reported to become pro-oxidant and contribute to oxidative stress. Under high concentrations (10^−4^–10^−2^ M), Tempol is known to contribute to oxidative stress [88, 89]. NAC also has a similar response and, in certain situations, can enhance the production of free radicals, such as ·OH [90]. Furthermore, it has been reported that some antioxidants can elicit a harmful effect on the differentiation of skeletal muscle progenitor cells. Ding et al. [91] reported that the inhibition of nuclear factor erythroid 2-related factor 2 (Nrf2), Nrf2-gultamate-cysteine ligase (GCL), and glutathione reductase (GR) through small interfering RNA (siRNA) blocked muscle differentiation in C2C12 cells. Lee et al. [92] reported that inhibition of complex I (a driver of O_2_·^−^ production) via siRNA, resulted in suppressed differentiation in H9c2 rat cardiac myoblasts. Moreover, this research was supported by Won et al. [93] who reported that peroxiredoxin-2 (Prx-2), which protects against oxidative stress, is upregulated during muscle differentiation, and treatment with NAC prevents Prx-2 upregulation, thus subduing myotube formation. Consequently, determining the correct balance between ROS level and antioxidant therapy is of great importance, as it is clear from what is discussed above, too much of either can have detrimental effects on bodily homeostasis.

## 7. Clinical Trials

Whilst there are many positives for the use of antioxidants, they have their limitations. There has been a small amount of studies investigating the effects of antioxidants on FSHD muscle and the potential benefits antioxidant therapies may have. Passerieux et al. [94] reported that dietary supplementation of vitamin C, vitamin E, zinc, and selenium did not improve the two-minute walk test in FSHD patients. However, at a mechanistic level, it did improve maximum voluntary contraction and endurance of the quadriceps. The contradictory findings may be due to the small population size of the study. In another study, van der Kooi et al. [95] reported that supplementation of folic acid and methionine (which can lead to an increase in DNA methylation) had no effect on DNA methylation in FSHD patients and subsequently showed no positive effect on the disease state or muscle health. More rigorous research is needed therefore before a conclusion can be made on the benefits of antioxidant supplementation in FSHD. Whilst the in vitro research is showing positive results, it would be naïve to extend conclusions to clinical settings.

With the antioxidant effects of exercise being noted previously, it is interesting to examine the literature on exercise interventions in FSHD. Bankole et al. [96] reported that a combined strength and interval cycling exercise-training program resulted in significant increases (*P* < 0.05) in VO_2_ peak, mean arterial pressure, muscle endurance, maximum voluntary contraction of the quadriceps, and 6-minute walking distance after 24 weeks. In addition, the amount of fatigue the patient experienced decreased (*P* < 0.01) with no exacerbation of muscle tissue damage. It is clear from the literature that exercise can be a potent antioxidant and can also elicit positive results in FSHD individuals, however, is it unclear whether antioxidant treatment would be beneficial or detrimental to the skeletal muscle adaptations in response to exercise [97]. It would be interesting to investigate the effects a combination of antioxidant and exercise therapy could have on FSHD muscle as this may shed light on whether antioxidants have a positive or negative effect on the exercise-induced skeletal muscle adaptations.

## 8. Conclusion

In conclusion, there is a vast amount of research which shows the beneficial effects antioxidants can have on reducing oxidative stress and associated cell damage. Whilst this research is primarily positive, there exists enough contradictory data to highlight that our understanding of what determines when antioxidants are beneficial or detrimental is not yet completely understood. Even less well understood are the effects of antioxidants on the disease process of FSHD. Current evidence suggests that antioxidants should suppress the oxidative stress response in myoblasts, thereby helping to preserve muscle quality and function. Overall, the results discussed throughout this review are strong indicators that supplementation of antioxidants may be beneficial for improving muscle health, particularly the functional and contractile properties in addition to alleviating some of the oxidative stress witnessed within FSHD patients; however, as of yet, there have been no large clinical trials that have been successful.
